# Obesity Challenge Drives Distinct Maternal Immune Response Changes in Normal Pregnant and Abortion-Prone Mouse Models

**DOI:** 10.3389/fimmu.2021.694077

**Published:** 2021-06-09

**Authors:** Yanhong Li, Jiajia Chen, Yikong Lin, Ling Xu, Yifei Sang, Dajin Li, Meirong Du

**Affiliations:** ^1^ Laboratory for Reproductive Immunology, NHC Key Lab of Reproduction Regulation (Shanghai Institute of Planned Parenthood Research), Shanghai Key Laboratory of Female Reproductive Endocrine Related Diseases, Hospital of Obstetrics and Gynecology, Fudan University Shanghai Medical College, Shanghai, China; ^2^ Department of Obstetrics and Gynecology, Guangzhou First People’s Hospital, School of Medicine, South China University of Technology, Guangzhou, China

**Keywords:** maternal obesity, normal pregnancy, spontaneous miscarriage, maternal immunity, overcompensation

## Abstract

Obesity is prevalent among women of reproductive age and is associated with increased risk of developing multiple pregnancy disorders. Pregnancy must induce immune tolerance to avoid fetal rejection, while obesity can cause chronic inflammation through activating the immune system. Impaired maternal immuno-tolerance leads to pregnancy failure, such as recurrent spontaneous abortion (RSA), one of the most common complications during early pregnancy. How does maternal immune response change under obesity stress in normal pregnancy and RSA? In turn, is obesity affected by different gestational statuses? Limited information is presently available now. Our study investigated pregnancy outcomes and maternal immune responses in two murine models (normal pregnancy and spontaneous abortion models) after obesity challenge with a high-fat diet (HFD). Abortion-prone mice fed HFD had significantly higher weight gains during pregnancy than normal pregnant mice with HFD feeding. Nonetheless, the embryo implantation and resorption rates were comparable between HFD and normal chow diet (NCD)-fed mice in each model. Evaluation of immune cell subsets showed HFD-induced obesity drove the upregulation of activated NK cell-activating receptor (NKp46)^+^ NK cells and pro-inflammatory macrophages (MHCII^high^ M*φ*) as well as CD4^+^ and CD8^+^ T cells in the normal pregnancy group. However, in the abortion-prone group, relative more immature NK cells with decreased activity phenotypes were found in obese mice. Moreover, there were increased DCreg (CD11b^high^ DC) cells and decreased CD4^+^ and CD8^+^ T cells detected in the HFD abortion-prone mice relative to those fed the NCD diet. Our findings reveal how pregnancy obesity and maternal immune regulation are mutually influenced. It is worth noting that the abortion-prone model where active maternal immune status was intensified by obesity, in turn stimulated an overcompensation response, leading to an over-tolerized immune status, and predisposing to potential risks of perinatal complications.

## Introduction

Obesity is recognized as a public health concern due to imbalanced energy metabolism and immune homeostasis attributable to overfeeding and sedentary lifestyles in modern society. Its prevalence is dramatically increasing worldwide, reaching pandemic standards in 2016 with 13% of adults being obese and 39% being overweight (1). Concomitant with the alarming incidence in both sexes, the growing obesity rates in women of child-bearing age deserve more attention given the associated high morbidity and mortality for both mother and offspring (2, 3). Indeed, obese women are nearly three times more likely to develop obstetric and perinatal complications during pregnancy (4), and emerging evidence has linked maternal obesity to multiple pregnancy-related disorders such as miscarriage, preeclampsia, preterm birth, and gestational diabetes (5–7). However, the mechanisms linking obesity and adverse pregnancy outcomes are poorly understood.

Among the underlying processes in maternal obesity thought to drive obstetric and perinatal complications, obesity-induced low-grade chronic inflammation has gained increasing attention (8–10). Excessive fat accumulation in obesity results from the overloading of adipocytes with triglycerides together with ectopic lipid deposition, resulting in the production of lipotoxic intermediates (11). In turn, immune cells infiltrate into adipose tissue, potentiating pro-inflammatory polarization and promoting the secretion of pro-inflammatory cytokines by leukocytes (12, 13). Supporting this notion, the peripheral blood of obese pregnant women contains elevated levels of tumor necrosis factor alpha (TNF-α), C-reactive protein (CRP), and interleukin 6 (IL-6) (14–16). The long-term presence of these pro-inflammatory factors not only induces systemic inflammation but also gives a rise to immune cell alterations at distal organ sites, leading to multi-organ dysfunction (17). Therefore, it is unsurprising that obesity-related stress alters the immune environment at the maternal–fetal interface, disrupting normal placental function which can lead to pregnancy failure (18). Several reported studies appear to verify this hypothesis. Perdu et al. showed that maternal obesity adversely affected vessel-to-lumen ratios in placental arteries which was associated with reduced numbers of uterine NK cells, shown to be indispensable for uterine artery remodeling and placental development (19). Similarly, Jennet et al. reported that the overall activity as well as the natural cytotoxicity receptor 1 (NCR1) expression level in uterine NK cells was increased in obese women, and these changes were tightly linked with dampened artery remodeling (20). Challier et al. found that obesity augmented the accumulation of pro-inflammatory macrophages in the placenta and induced inflammatory milieu at the maternal–fetal interface (21). Accordingly, maternal obesity influences the biology of diverse subsets of uterine immune cells, altering the immune cell landscape of the decidua.

As we know, the fetus, whose half antigens are inherited from the father, is immunologically recognized as a semi-allograft to the mother. Thus the maternal immune system is obligated to make active adaptations to tolerate the embryo for successful pregnancy maintenance (22). Unfortunately, once the maternal immune system fails to turn into immunosuppressive, the fetus would be attacked and here come diverse gestational complications, in which spontaneous abortion is the most common one of early pregnancy (23). Recurrent spontaneous abortion (RSA), defined as two or more consecutive spontaneous abortions before 20 weeks of gestation, is considered as a severer disease entity compared with sporadic miscarriage (23). Though genetic, structural, infective, and endocrine anomalies have been related to the incidence of RSA, the etiology of approximately 40% RSA is still unexplained and up to half of RSA with unknown causes are attributed to dysregulated maternal–fetal immunotolerance (24–26). Actually, the immune responses in RSA patients are transformed from an anti-inflammatory status towards an activated and pro-inflammatory direction. As stated above, obesity-associated inflammation has been reported to impair the pregnancy immunotolerance, increasing the risks of pregnancy loss (27, 28). Since inflammation *per se* is evidenced to be an important factor to cause and promote obesity, it is interesting to explore whether obesity and RSA would interact reciprocally in which maternal immune system may serve as a connecting bridge. And how would the maternal immunity respond to the combined challenges of immune-activated obesity and pro-inflammatory RSA? Answering these questions would help to ascertain the effects of two inflammation-driven events within an immunosuppression-required background, hoping to offer some clues for the explorations of novel mechanisms and therapies in obese pregnant women with a history of RSA.

To address these questions, we applied normal pregnancy (NP) and abortion-prone (AP) mice to investigate the pregnancy outcomes along with maternal immune responses under obesity stress. The abortion-prone mouse model (29), DBA/2-mated female CBA/J mice, is widely accepted as an immune-based model of human RSA not only for its guaranteed high rate of pregnancy loss (30), but also on account of plentiful inflammatory leukocytes extensively infiltrating into the maternal–fetal interface, which mimics human miscarriages with immune causes (31). This study helps to better understand the relationships and interactions among maternal immune status, maternal obesity, and embryo tolerance, hoping to generate logical and testable ideas for the management and treatment of overweight mothers with a history of RStA.

## Materials and Methods

### Animal Experiments

Female CBA/J and male and DBA/2 mice (8–10 weeks old) were purchased from Shanghai JieSiJie Laboratory Animal Co. Ltd. Male Balb/c mice (8–10 weeks old) were purchased from the Department of Laboratory Animal Science, Fudan University (Shanghai, China). Normal pregnancy models and the well-known abortion-prone models were established by CBA/J × Balb/c mating and CBA/J × DBA/2 mating (30, 31), respectively. Ten weeks before mating, female CBA/J mice were randomly divided into two groups and fed either with a regular chow diet or a highly palatable obesogenic diet consisting of high-fat pellets (HF; 99 40 kcal%; Research Diets, New Brunswick, NJ, USA). NCD-fed female CBA/J mice were randomly divided into two groups (n = 25/group) and mated in natural cycling with male Balb/c mice or DBA/2 mice, respectively, with the presence of a vaginal plug chosen to indicate day 0.5 of gestation. HFD-fed female CBA/J mice were subjected to the same protocol (n = 25/group). Dams were weighed weekly before and during gestation and on GD12.5, female CBA/J mice along with the unborn fetuses were sacrificed to examine the fetal resorption rate and placenta weight. Blood plasma sampling, uteri, spleens, and inguinal lymph nodes were collected for immune cell isolation. All experimental procedures involving animals were conducted in accordance with the Guide for the Care and Use of Laboratory Animals (China), with approval from the Human Research Ethics Committee of Obstetrics and Gynecology Hospital of Fudan University.

### Isolation of Decidual Immune Cells and Peripheral Blood Mononuclear Cells

Uteri from pregnant mice were carefully dissected free from the mesometrium and separated from the ovaries and cervix. The dissected uteri were washed twice in ice-cold PBS and cut into 1 mm^3^ pieces before enzymatic digestion in DMEM F12 medium supplemented with 1 mg/ml type IV collagenase and 0.2 mg/ml DNase I (Sigma-Aldrich Corp., St. Louis, MO, USA) for 40 min at 37°C with gentle agitation. The suspension was filtered through a 70-μm cell strainer and enriched by discontinuous Percoll gradient centrifugation (GE Healthcare Life Sciences, Little Chalfont, UK). The decidual immune cells obtained from the 40%/60% Percoll interface and peripheral blood mononuclear cells (PBMCs) isolated using Ficoll density gradient centrifugation at 800×*g* for 20 min were washed twice in PBS before analysis.

### Preparation of Spleen Cells and Lymph Node Cells

Isolated murine spleen tissues and inguinal lymph nodes were aseptically stored in RPMI 1640 medium before processing. To produce single-cell suspensions, spleen tissue was passed through a 70-μm mesh strainer using a 10 ml syringe plunger while the inguinal lymph nodes were pushed through a 40-μm cell strainer. Cells were then washed with PBS and cultured in RPMI 1640 medium 10% containing fetal bovine serum, 100 U/ml penicillin, and 100 mg/ml amphotericin B at 37°C in 5% CO_2_.

### Flow Cytometry

Cell surface staining was conducted with the appropriate fluorochrome-conjugated Abs for 30 min at 4°C. The following specific monoclonal antibodies (mAbs) were used: antigen-presenting cells (APC)-cy7-conjugated anti-mouse CD45, FITC-conjugated anti-mouse CD3, Percy5.5-conjugated anti-mouse CD4, FITC-conjugated anti-mouse CD8, PEcy7-conjugated anti-mouse NK1.1, BV421-conjugated anti-mouse Nkp46, BV421-conjugated anti-mouse major histocompatibility complex II (MHC-II), APC-conjugated anti-mouse F4/80, PE-conjugated anti-mouse CD11c, PE-CF594-conjugated anti-mouse CD11b and APC-conjugated anti-mouse CD27 (all purchased from BD Bioscience). Flow cytometric (FCM) analyses were performed on a CyAn ADP analyzer (Beckman Coulter, Inc., Kraemer Boulevard Brea, CA, USA), with data analyzed using FlowJo Version 6.1 software (TreeStar, Asland, OR, USA).

### Statistical Analysis

Prism 6 software (GraphPad) was used for data analysis. Statistical significance was determined using Student’s t-test for two-group or one-way ANOVA for multiple group comparisons. The data are presented as mean ± SEM. Statistical significance was defined as P < 0.05.

## Results

### Effects of HFD on Weight Gain and Pregnancy Outcomes in Normal and Abortion-Prone Mouse Models

**Figure 1:**
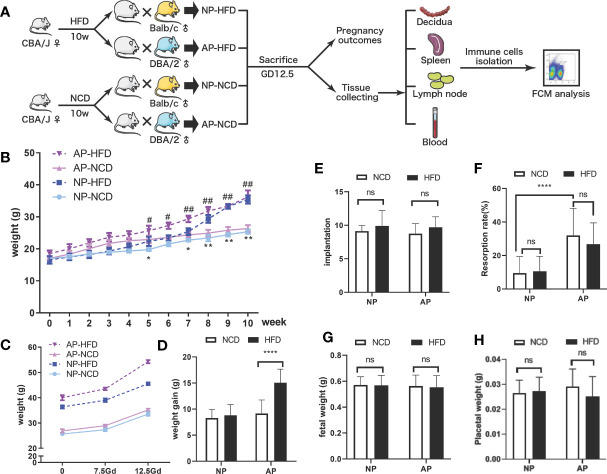
Effects of HFD on weight gain and pregnancy outcomes in normal and abortion-prone mouse models. **(A)** Schematic diagram showing the experimental design of the mouse models involving the establishment, gestational termination at GD12.5, pregnancy outcomes observation, immune cell isolation and analysis. Mouse weights during normal pregnancy (NP, dark and light blue) or spontaneous abortion (AP, dark and light purple) were measured from start of the experimental diet, on either a high-fat diet (HFD, dotted line) or a normal chow diet (NCD, solid line) before mating **(B)** and during gestation **(C)**. **(D)** Maternal weight increments during gestation on GD12.5. All dams were killed on GD12.5, and the implantation numbers **(E)**, resorption rates **(F)**, fetal weights **(G)** as well as placental weights **(H)** were presented. Data are presented as mean ± SEM. NS, not significant; *p < 0.05, **p < 0.01, ****p < 0.0001, ^#^p < 0.05, ^##^p < 0.01.

To investigate maternal obesity and pregnancy outcomes, we established an obese mouse model by feeding female CBA/J mice with high-fat pellet diets for 10 weeks before mating, while control mice were fed a regular chow diet (**Figure 1A**). Mice fed the HFD gained approximately three-fold more weight than the control diet group, indicative that the feeding regimen induced an obese state (**Figure 1B**). To explore the influence of maternal obesity as well as immune responses on pregnancy outcomes, the obese and control mice were randomly divided into two groups, respectively, and mated with Balb/c or DBA/2 male mice to construct normal (NP) and abortion models (AP). As expected, the pregnant dams from all four groups displayed weight gains during gestation (**Figure 1C**). Among the groups, the AP-HFD group had the greatest weight gain, reaching a significant difference at GD12.5 compared with the AP-NCD group (**Figure 1D**). However, the weight gains during gestation in NP-NCD and NP-HFD groups were comparable.

Pregnant mice were then sacrificed on GD12.5 to observe pregnancy outcomes. There were no significances observed in the embryo implantation numbers in NP and AP models fed either HFD or NCD (**Figure 1E**). However, consistent with previous reports (32, 33), resorption rates for the NP model were about 10% which increased to about 30% in the AP model, which was not obviously influenced by HFD or NCD (**Figure 1F**). Moreover, comparable fetal and placental weights were observed in both NP and AP mice irrespective of the feeding regimen (**Figures 1G, H** **)**. Thus, the pregnancy outcomes showed no obvious difference between HFD- and NCD-fed mice in NP and AP models, but the AP model treated with HFD had greater weight gain during gestation.

### Maternal Obesity Differentially Alters NK Cells in Mice With Normal Pregnancy or Spontaneous Abortion

**Figure 2:**
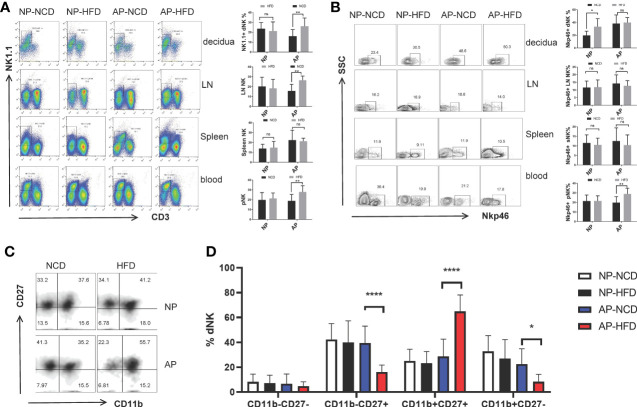
Maternal obesity exerts differential effects on NK cells in NP and AP mice. On gestational day 12.5 (GD12.5), cells from deciduas, lymph nodes (LN), spleens and blood were collected and analyzed by flow cytometry. **(A)** Representative and quantitative results for NK cells in the indicated tissues derived from NP and AP models with NCD or HFD fed. **(B)** Representative and quantitative results for NKp46 expression in NK cells. **(C)** Representative flow cytometry results for CD27 and CD11b expression in decidual NK cells. **(D)** Proportions of CD11b^−^CD27^−^, CD11b^−^CD27^+^, CD11b^+^CD27^+^ and CD11b^+^CD27^−^ NK cell subsets in the deciduas. Data are presented as mean ± SEM. NS, not significant; *p < 0.05, **p < 0.01, ****p < 0.0001.

To characterize the influence of maternal obesity on NK cells, we analyzed the frequencies and phenotypes of NK cells isolated from maternal decidua, inguinal lymph nodes, spleens, and peripheral blood by flow cytometry. Compared to the AP-NCD group, except for splenic NK cells, local (including decidua and inguinal lymph nodes) and circulatory NK cells (peripheral blood) in the AP-HFD group were significantly increased. Nonetheless, there were no obvious differences in the proportion of NK cells in the NP groups between HFD- and NCD-fed mice (**Figure 2A**).

NKp46 is a marker of NK cell activation and participates in the modulation of NK cell cytotoxicity as well as cytokine production. Several reports have associated NKp46 with decidual angiogenesis and uterine vascular remodeling during pregnancy (34–36). Therefore, to determine whether maternal obesity affected NK cell activity, we examined NKp46 expression. Notably, the HFD upregulated the percentage of NKp46^+^ NK cells in the decidua of the NP group and peripheral blood of the AP group, but had no influence on other sample comparisons (**Figure 2B**).

Alternatively, to evaluate the effect of maternal obesity on NK cell maturation, we used staining of the surface markers CD11b and CD27 to subdivide NK cells into four subsets coinciding with four stages of the NK cell maturation process, namely CD11b^−^CD27^−^ → CD11b^−^CD27^+^ → CD11b^+^CD27^+^ → CD11b^+^CD27^−^ (37–39). Analysis of decidual NK (dNK) cells showed the AP-HFD group displayed lower proportions of CD11b^−^CD27^−^ cells (although not significant) and the CD11b^−^CD27^+^ and CD11b^+^CD27^−^ subsets, but a prominently higher proportion of the CD11b^+^CD27^+^ subset compared with AP-NCD group (**Figures 2C, D** **)**. These changes were not observed in the NP groups either fed with NCD or HFD, nor in NK cells isolated from lymph nodes, spleen, or blood (**Supplementary Figure 1**). This indicates there is a specific inhibitory effect on dNK cell maturation in the HFD mice in the AP but not in NP models. Furthermore, this implies that maternal obesity drives dNK cell differentiation toward relatively mature CD11b^+^CD27^+^ subset but prevents its further maturation from CD11b^+^CD27^+^ to CD11b^+^CD27^−^ subset at the maternal–fetal interface in spontaneous abortion mice.

Collectively, these data demonstrate that NK cells from the NP and AP models have different responses to obesity challenge. Specifically, a high-fat diet leads to increased NK cell activity, but the number of NK cells remains unchanged in normal pregnancy. In contrast, the HFD augments NK cells with a less mature phenotype in abortion prone mice.

### Distinct Responses of Myeloid Cells in Normal Pregnancy and Abortion-Prone Mouse Models to Maternal Obesity

**Figure 3:**
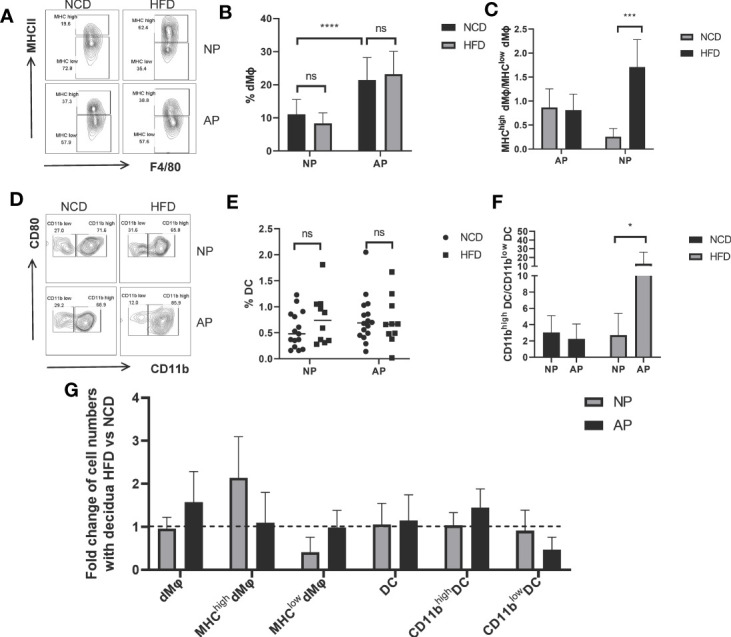
Different responses of myeloid cells to maternal obesity challenge in NP and AP models. **(A)** Representative flow cytometric analysis of macrophages isolated from deciduas stained with MHC-II and F4/80 fluorochrome-conjugated antibodies. **(B)** Total numbers of decidual macrophages. **(C)** Ratios of MHC-II^high^ *versus* MHC-II^low^ macrophage subsets in deciduas. **(D)** Representative flow cytometry analysis of DCs within pregnant uteri marked with CD80 and CD11b fluorochrome-conjugated antibodies. **(E)** Total numbers of decidual DCs. **(F)** Ratios of CD11b^high^ *versus* CD11b^low^ DC subsets in deciduas. **(G)** Summary of fold changes in decidual myeloid immune cell numbers under obesity challenge in NP and AP models. Data are presented as mean ± SEM. NS, not significant; *p < 0.05, ***p < 0.001, ****p < 0.0001.

To further investigate the impact of maternal obesity on decidual myeloid cells, the frequencies of decidual macrophages and DCs along with their specific subsets were analyzed. The number of macrophages identified by F4/80 and MCH-II markers showed no significant differences between HFD and NCD groups both for the NP and AP mouse models (**Figures 3A, B** **)**. Nevertheless, as expected, the abundance of total macrophages in the AP group was markedly higher than in the NP group (**Figures 3A, B** **)**. Next, we compared the percentages of pro-inflammatory macrophage subsets (MHC-II^high^ M*φ*) in the decidua among the four groups. Instructively, HFD-induced obesity upregulated the ratio of MHC-II^high^ M*φ* to MHC-II^low^ M*φ* in the NP group, whereas the pro-inflammatory and anti-inflammatory macrophages were comparable between HFD and NCD groups in AP mice (**Figure 3C**).

With respect to total decidual DCs, there were no significances between NP and AP models fed with NCD or HFD (**Figures 3D, E** **)**. However, we also analyzed CD11b expressing DCs which represent a specialized subset called regulatory DCs (DCregs) that function to inhibit immune responses similar to Tregs (40, 41). To investigate whether immunosuppressive DCs were altered following maternal obesity or the abortion-prone state, we subdivided decidual DCs using CD11b to calculate the ratio of CD11b^high^ DCs to CD11b^low^ DCs. Indeed, we observed this ratio was dramatically increased in the AP-HFD group compared to the other groups (**Figures 3D–F**), indicative that immunosuppressive DCs are only altered in mice where the effects of maternal obesity and spontaneous abortion are superimposed.

The different responses of myeloid cells derived from NP or AP models to maternal obesity are summarized in **Figure 3G**. As depicted, the HFD has no influence on the total amount of decidual macrophages but increases the proportion of the MHC-II^high^ M*φ* subset, while the inverse effects occur in the AP models. Regarding DCs, comparable DC numbers together with the proportions of DCs subsets were observed in NP mice with or without HFD. In contrast, although the total DC numbers are unchanged in AP mice fed the HFD, there is an increased proportion of DCregs. Thus, the myeloid cells in NP exhibit an enhanced inflammatory response under the pressure of maternal obesity, while the myeloid cells in AP unexpectedly exhibited a state of immunosuppression.

### Maternal Obesity Augments T Cells in Mice With Normal Pregnancy but Suppresses T Cells in Spontaneous Abortion Mice

**Figure 4:**
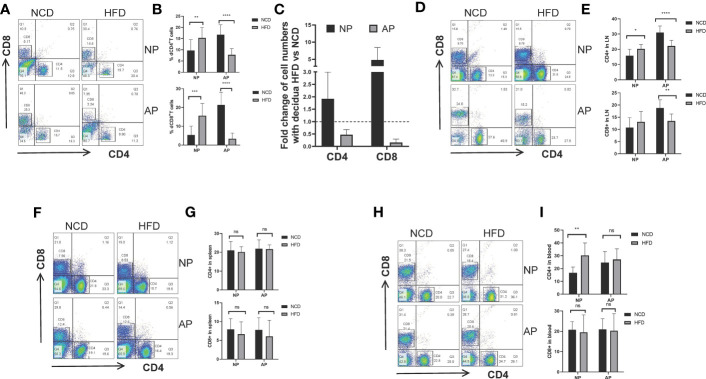
Maternal obesity mediates opposite effects on CD4^+^ and CD8^+^ T lymphocytes in NP and AP models. **(A, B)** Representative and quantitative flow cytometry results for CD4^+^ and CD8^+^ T cells in deciduas derived from NP and AP models fed with NCD or HFD. **(C)** Fold changes of decidual T cell numbers in NP and AP mice under obesity challenge. **(D, E)** Frequencies of CD4^+^ and CD8^+^ T cells in lymph nodes. **(F, G)** Frequencies of CD4^+^ and CD8^+^ T cells in spleens. **(H, I)** Frequencies of CD4^+^ and CD8^+^ T cells in blood. Data are presented as mean ± SEM. NS, not significant; *p < 0.05, **p < 0.01, ***p < 0.001, ****p < 0.0001.

To evaluate the potential effect of high-fat intake on maternal adaptive immune cells during pregnancy, we examined the proportions of CD4^+^ and CD8^+^ T cells collected from the maternal decidua, uterine-draining lymph nodes, spleens, and peripheral blood. For NP mice there were increased decidual CD4^+^ and CD8^+^ T cells in the HFD group compared to the NCD group. Conversely, the numbers of decidual CD4^+^ and CD8^+^ T cells were comparatively decreased in AP mice fed the HFD (**Figures 4A–C**). Similar findings were evident in CD4^+^ and CD8^+^ T cells derived from lymph nodes (**Figures 4D, E** **)**. However, no significant differences involving T lymphocytes were observed in the spleen or peripheral blood between the HFD and NFD groups in either pregnancy model, except for increased blood CD4^+^ T cells in NP-HFD mice compared with NP-NCD mice (**Figures 4F–I**).

These data suggest that with the stimulation of high-fat diet, maternal adaptive immune cells were augmented in NP models but were unexpectedly diminished in AP models.

### Pre-Gravid Obesity Changes Affect the Maternal Immune System Differently in Normal Pregnancy and Spontaneous Abortion States

**Figure 5:**
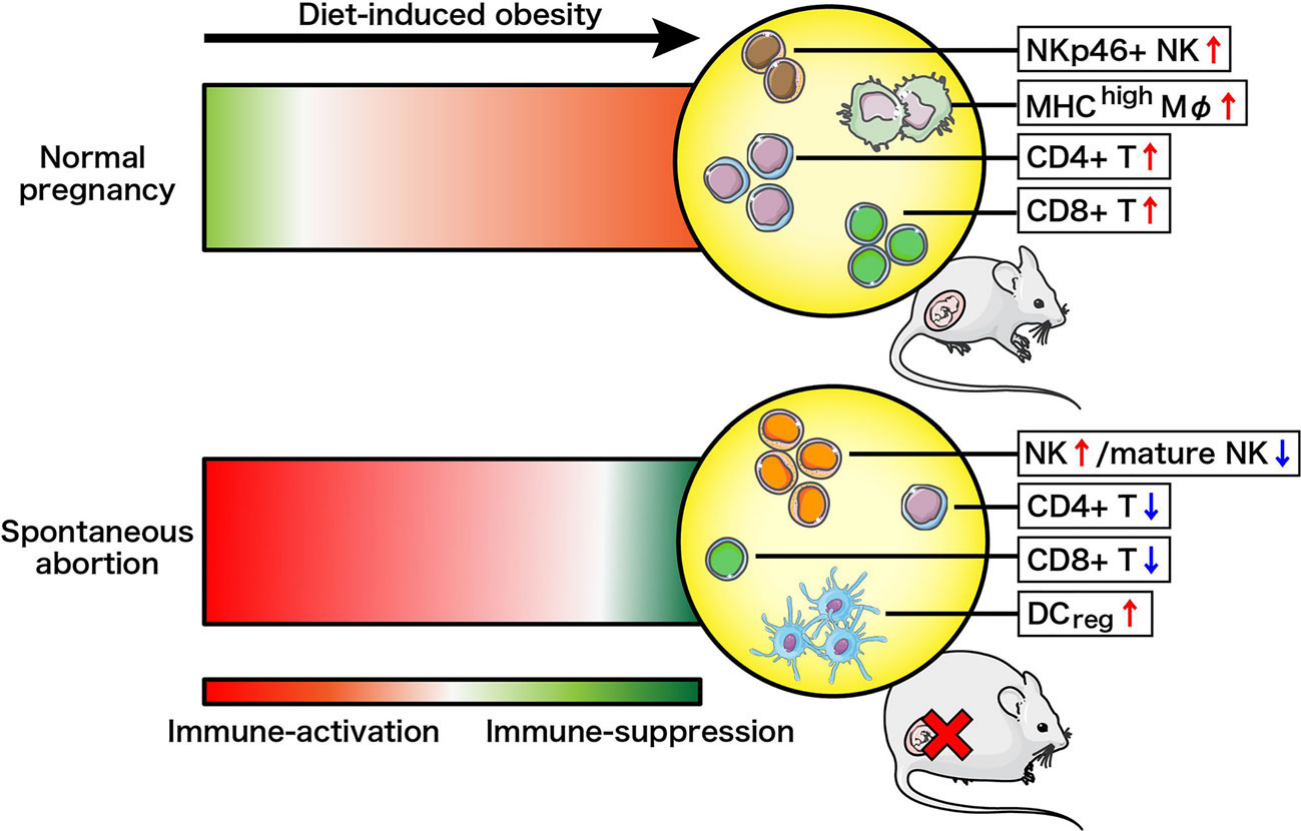
Schema of how the maternal immune system changes driven by diet-induced obesity in normal pregnancy or spontaneous abortion. Immune-suppression and immune-activation changes are illustrated by red and green, respectively, with darker color changes reflecting more drastic changes.

We constructed a model to illustrate the changes in immune cell landscapes resulting from diet-induced obesity and different gestational status (**Figure 5**). Pregnancy is immunologically defined by the active adaptation of the maternal immune system towards immunosuppression to prevent fetal rejection, especially at the maternal–fetal interface (42, 43). Here we defined a phenotypic and numerical switch of overall immune cells from suppression towards activation following high-fat intake in NP mice, supported by the upregulation of decidual NKp46^+^ NK, MHC-II^high^ M*φ*, CD4^+^ T and CD8^+^ T cells. Conversely, the maternal immune system is generally acknowledged as severely immune-activated in cases of spontaneous abortion (23, 44). Surprisingly, our study demonstrates that pre-gravid obesity drove the deepening of immunosuppression instead of exacerbating the pre-existing inflammation in AP mice. We recorded decreased proportions of mature cytotoxic NK cells, CD4^+^ T cells as well as CD8^+^ T cells in the decidua, although there was local and systemic augmentation of total NK cells along with uterine DCregs observed in obese abortion-prone mice. The immune system changes driven by maternal obesity in NP and AP mice are therefore very distinct.

## Discussion

Using murine models of normal and abortion-prone pregnancy, our study investigated the relationships among maternal obesity, pregnancy outcomes, and maternal immune responses by examining the frequencies along with phenotypes of innate and adaptive immune cells both locally within the uterus and systemically. We found that spontaneous abortion aggravated obesity in gestational obese mice compared with normal pregnancy, but nevertheless pre-gravid obesity did not pose serious consequences for reproductive health either in the NP or AP groups. Among the key alterations observed in immune cells, HFD-induced obesity upregulated decidual NKp46^+^ NK cells, decidual MHC-II^high^ pro-inflammatory macrophages and T lymphocytes derived from deciduas, lymph nodes as well as peripheral blood in NP mice. While in the AP mouse model, maternal obesity drove overall increases in dNK cells along with functional changes represented by the intensification of the CD11b^+^CD27^+^ subset. Suppressive DCregs were also amplified in the decidua, accompanied by decreased CD4^+^ and CD8^+^ T cell numbers within the uterus. Together these observations provide new insights into how gestational status and maternal obesity are mutually influenced and delineate how low-grade systemic inflammation induced by obesity is superimposed onto the pregnant immune state during normal or under spontaneous abortion conditions. To our knowledge, this is the first research involving abortion-prone mouse models in the investigation of interactions between pre-pregnancy obesity and maternal immune responses.

Many previous studies have evaluated the reciprocal connections between maternal obesity and pregnancy outcomes. Although obesity-induced mal remodeling of decidual spiral arteries has been substantiated (19, 20), pregnancy outcomes such as modes of delivery, gestational ages, implantation sites, placental weights, and litter sizes remain unchanged (11, 15, 20, 45), which is in line with our findings. However, studies investigating the effects of pregnancy on maternal obesity are scarce, especially when it comes to pathological states such as miscarriage. Ingvorsen et al. reported that an obesogenic diet increased macrophage counts in mouse adipose tissues and livers, but gestation reversed macrophage infiltration in lipometabolic-related organs, thus attenuated the impact of obesity-induced inflammation (11). Nonetheless, we did not detect any weight gain differences during normal pregnancy, while interestingly there were significantly increased weight gains observed in the obese AP mice. These results indicate that the inflammatory environment caused by spontaneous abortion may in turn affect the systemic fat metabolism, thus sharpening the influence of adipose tissues under obesity challenge.

Among immune cell subsets at the maternal–fetal interface, NK cells are the most abundant, accounting for ~70% in humans and ~35% in mice (46, 47). NK cells are indispensable in maintaining normal pregnancy and play an innate sentinel role in peripheral tissues during pregnancy (48). The results of our analysis of NK cells were partly concurrent with previous studies (15, 20, 49). We found obesogenic diets did not alter the frequencies of NK cells either locally or systemically in NP mice, although a significant reduction in uterine NK cell numbers as well as decreased proportions in the uterus-draining lymph nodes were reported by others (19, 45). However, the results from AP mice were different from NP mice where obesity increased the percentages of NK cells both in the decidua and in circulation. To our knowledge, the uterine and peripheral NK cells show a tendency to increase in RSA (50, 51), while the reports of obesity-driven alterations in NK cell abundance are often conflicting (1). This highlights the intricacy of immunoregulation in obesity, which is further complicated by the maternal immune response. With respect to our findings in the AP mice, we propose that pre-gravid mice may undergo global low-grade chronic inflammation resulting in the maternal immune system becoming pre-activated, whereas in NP, NK cells remain unaltered because of effective compensatory mechanisms to prevent changes. Nevertheless, the double challenges of obesity and miscarriage stimulate the over-proliferation of NK cells during AP gestation. However, whether the expansion of dNK cells comes from infiltrating peripheral NK cells or the proliferation of tissue resident NK cells at the maternal–fetal interface needs further exploration.

To examine NK cell function, we utilized biomarker analysis of NKp46 and CD11b combined with CD27 to interpret NK activity and maturation, respectively. Prior research has confirmed the effects of pregnancy and obesity on the expression of NKp46, that is, normal pregnancy upregulates while spontaneous abortion and obesity downregulates the frequencies (49, 52–55). Research investigating the combined impacts of these two events is lacking, although recently, Jennet et al. reported that pregnancy increased the proportion of decidual NKp46^+^ NK cells in obese pregnant mice (20). Consistently we found that the high-fat diet induced the expression of NKp46^+^ dNK cells in NP mice without affecting peripheral NK cells. However, opposing findings were made in AP mice where there were increased NKp46^+^ NK cells in the periphery but unaltered numbers in the decidua. The possible explanation may involve the degree of balance between the processes involved: normal pregnancy upregulates circulating NKp46^+^ NK cells which is counteracted by the effects of maternal obesity whereas in spontaneous miscarriage, the maternal immune system overcompensates under the merged threats of abortion with obesity to maintain successive gestations. This in turn, augments NKp46^+^ NK cells which create a pregnancy-protective environment (34, 35). Furthermore, the NK cell maturation state was also changed between AP and NP mice, with obesity combined with AP driving the dNK cell differentiation from immature CD11b^−^CD27^−^ or CD11b^−^CD27^+^ to relatively mature CD11b^+^CD27^+^ phenotypes and prevented further maturation into the CD11b^+^CD27^−^ subset. Changes in the expression profiles of CD11b and CD27 can also be used a proxy measures of NK cell function in terms of cytokine secretion and cytolytic activity, respectively. Prior functional analyses have shown the CD27^−^CD11b^−^ subset displays immune tolerance behavior; CD11b^−^CD27^+^ as well as CD11b^+^CD27^+^ subsets exhibit more efficient cytokine production and the CD11b^+^CD27^−^ subset demonstrates the highest cytolytic function (37). In light of this, the alterations we observed in dNK cell maturity can be extrapolated such that the NK cell subsets in AP-HFD mice display enhanced cytokine secretion but weakened cytotoxicity compared to their NCD counterparts, which would benefit the maintenance of pregnancy. Based on this interpretation, we propose an “overcompensation hypothesis” to explain our findings which involve the overcompensation of the maternal immune system to the combined effects of obesity and abortion.

The second most abundant leukocytes at the maternal–fetal interface are decidual myeloid cells including macrophages and DCs (56). DCs are potent antigen presenting cells which function to bridge the innate and adaptive immune systems (57, 58). To further investigate the maternal immune response alterations, we quantified uterine macrophages as well as DCs. At least partially consistent with the findings of previous studies (11, 45), we found that the frequencies of either macrophages or DCs did not vary regardless of whether the gestational mouse models were obese or lean. However, other research showed increases in pro-inflammatory macrophages along with the decreased abundance of plasmacytoid DCs in pregnant obese subjects (12, 15, 21), indicating that delicate changes may occur in myeloid cell subsets rather than gross changes in total numbers. Therefore, we refined our analysis to include assessment of pro-inflammatory and anti-inflammatory profiles of decidual macrophages as well as DCs. Surprisingly, the percentages of MHC-II^high^ pro-inflammatory macrophages in NP and CD11b^high^ immunosuppressive DCs in AP were upregulated dramatically in mice fed with obesogenic diets, while MHC-II^high^ macrophages isolated from AP mice were not increased. Together these data provide the conclusion that maternal obesity promotes the local inflammation in the decidua of normal pregnancy but polarizes the immune environment of abortive mice into a condition of immunological depression, consistent with the “overcompensation hypothesis”.

Regarding the adaptive immune system, there is no general consensus about the impact of maternal obesity on CD4^+^ or CD8^+^ T cell numbers in pregnant females in prior literature (15, 45, 59–61). Nonetheless, multiple studies have demonstrated that obesity affects T cell phenotype and behavior, leading to increased ratios of central memory and effector memory T cells (15, 62), reduced proportions of naïve T cells and enhanced polarization of T cells towards Th1/Th17 subsets (10, 15). More importantly, one remarkable characteristic of obese stimulation is persistence, with long-term obesity resulting in T cell exhaustion and thymus aging (63). In this study, we showed that pre-gravid obesity upregulated CD4^+^ or CD8^+^ T cell frequencies in NP while downregulated T lymphocyte abundance in AP mouse models. This proposes that maternal obesity induces T cell proliferation in NP but inhibits the activation of adaptive immune cells in AP mice, indicative of overcompensation mechanisms in the obese abortive subjects. However, tempering this interpretation, our study did not measure T cell function, and it remains unclear whether decreases in CD4^+^ and CD8^+^ T cell numbers truly result from “overcompensation” or whether T cell exhaustion following chronic inflammation resulting from pre-pregnancy obesity may have caused these changes.

It is worth noting that our experimental samples were collected from GD12.5 mice, corresponding to the second trimester in which the maternal immune system becomes optimally tolerized (42), thus the model was well placed to study the effects of obesity on immune tolerance during pregnancy. Nevertheless, Jennet et al. demonstrated that maternal obesity disrupted placental vascular remodeling in early–mid gestation, but proper artery remodeling was observed by mid–late gestation (20). This indicates the maternal immune response is dynamic in nature, and gestational day effects must be considered when determining the effects of obesity on immune responses.

Finally, looking at our findings from the translational perspective, clinical research has tightly linked obesity in women with the increased incidence of genital tract infections, urinary tract infections, and wound infection along with a high prevalence of spontaneous miscarriage (64–66). However, the underlying connections between these phenomena are undefined. Our study provides a possible explanation that women with obesity suffer from the systemic low-grade chronic inflammation and are liable to abort, thus the maternal immune system switches to an immunosuppressive state according to the “overcompensation hypothesis”, leading to decreased resistance to foreign pathogens and increased risk of infections as observed.

In conclusion, our study determined the interactions between maternal obesity and pregnancy outcomes and reported the global immune cell landscape alterations under the challenge of obesity with normal pregnancy or spontaneous abortion. To explain the results, we propose a new “overcompensation hypothesis” in which the maternal immune response was inhibited, leaving potential risks of perinatal infection, suggesting that much attention should be paid to prevent infectious diseases during pregnancy and the perinatal period in overweight mothers with a history of RSA. Moving forward, investigations into the precise mechanisms of the mutual effects of maternal obesity and embryo tolerance as well as studies to address the “overcompensation hypothesis” will be of great importance.

## Data Availability Statement

The datasets presented in this study can be found in online repositories. The names of the repository/repositories and accession number(s) can be found in the article/**Supplementary Material**.

## Ethics Statement

The animal study was reviewed and approved by the Human Research Ethics Committee of Obstetrics and Gynecology Hospital of Fudan University.

## Author Contributions

YhL took the lead in planning the specific research, performing experiments, and generating all the figures. JC researched literatures and wrote the first draft of the manuscript. YkL helped to carry out the experiments and finish this article. LX and YS examined and provided critical feedback for the manuscript. DL took part in the project conception. MD was responsible for the conception, design of this study, and revision of the manuscript. All authors contributed to the article and approved the submitted version.

## Funding

The work is supported by the National Key R&D Program of China (2017YFC1001403), the National Nature Science Foundation of China (81630036, 31970859, 31900663), International cooperation project between Macao and Shanghai Municipal Commission of science and technology (20410760300), and The Strategic Collaborative Research Program of the Ferring Institute of Reproductive Medicine Supported by Ferring Pharmaceuticals and Chinese Academy of Sciences (FIRMX200504).

## Conflict of Interest

The authors declare that the research was conducted in the absence of any commercial or financial relationships that could be construed as a potential conflict of interest.
