# Supplementary figures and images for: Obesity Challenge Drives Distinct Maternal Immune Response Changes in Normal Pregnant and Abortion-Prone Mouse Models
Source: Front Immunol. 2021 Jun 9;12:694077. doi: 10.3389/fimmu.2021.694077 (PMC8219966; doi:10.3389/fimmu.2021.694077)

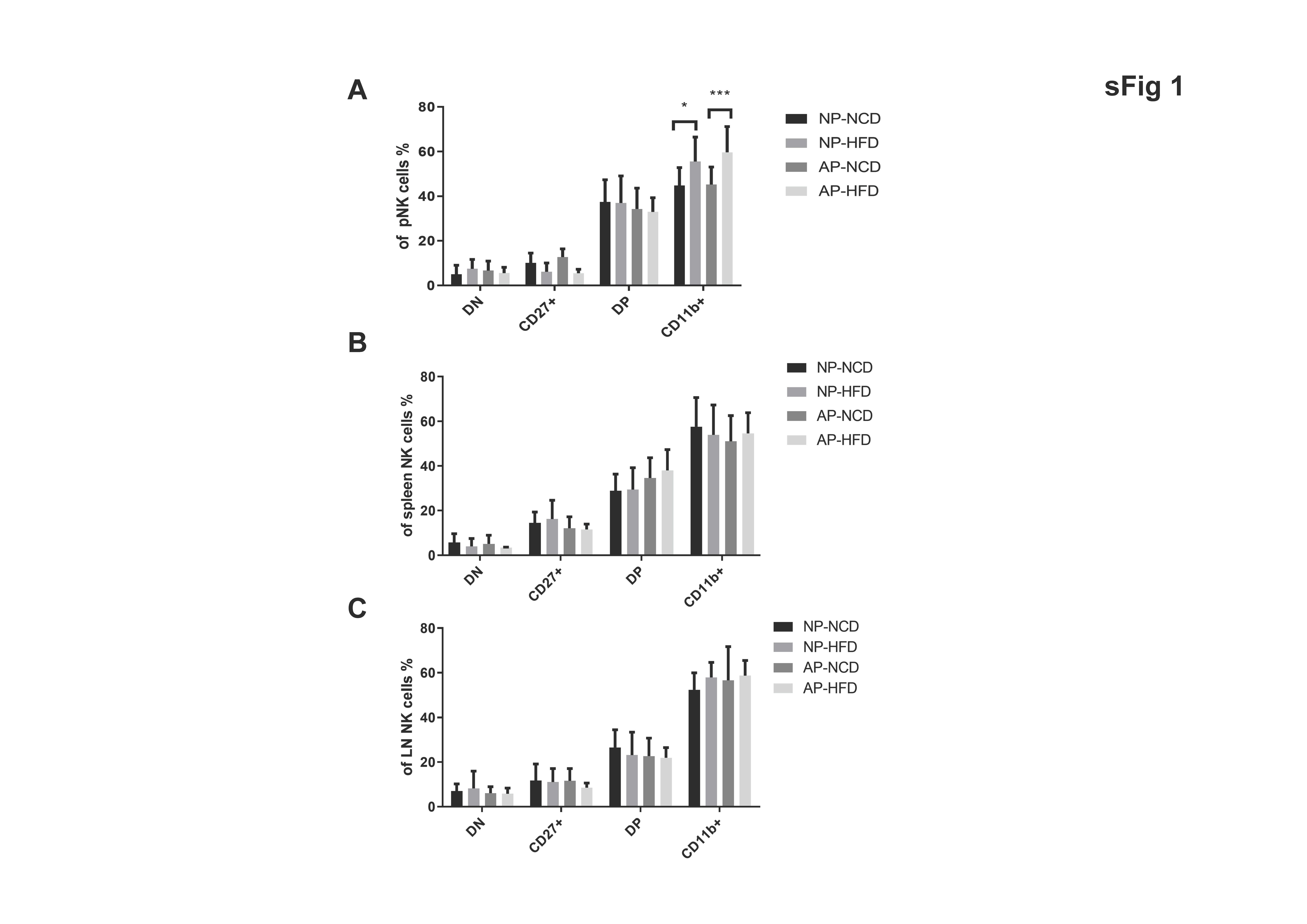

Supplement: Supplementary file 1 [file Image_1.tiff]
